# A highly conserved basic motif in the wing domain of portal protein is necessary for oligomerization and incorporation in phage P22

**DOI:** 10.1128/jvi.00133-26

**Published:** 2026-06-12

**Authors:** Makayla N. Leroux, Lohra M. Young, Martin F. Jarrold, Carolyn M. Teschke

**Affiliations:** 1 Department of Molecular and Cell Biology, University of Connecticut 124501 , Storrs, Connecticut, USA; 2 Megadalton Solutions, Bloomington, Indiana, USA; 3 Department of Chemistry, Indiana University 248013, Bloomington, Indiana, USA; 4 Department of Chemistry, University of Connecticut 7712, Storrs, Connecticut, USA; Michigan State University, East Lansing, Michigan, USA

**Keywords:** scaffolding protein, portal protein, DNA packaging, virus assembly

## Abstract

The dodecameric portal ring complex plays a critical role in the assembly of most double-stranded DNA (dsDNA) viruses and bacteriophages. The fidelity of ring oligomerization and incorporation of a single 12-mer portal ring is driven by interactions with a viral scaffolding protein. Structural evidence indicates that the wing domain of the portal is a crucial site for scaffolding protein interactions. In this study, the functional significance of the highly conserved _272_KRRR_275_ motif in the portal wing domain of bacteriophage P22 is investigated. Alteration of the entire motif to alanines results in a reduction in infectious phage production owing to a decrease in portal incorporation into procapsids *in vivo*. Further interrogation of the variant portal protein *in vitro* showed that it assembles into oligomeric assemblies larger than dodecamers, as determined by charge detection mass spectrometry. Though variant monomers have an altered secondary structure, they are still capable of assembling into rings in the presence of scaffolding protein, but it is likely that these rings adopt a modified conformation that is unfit for incorporation. These findings indicate that the _272_KRRR_275_ motif is necessary for critical intra-subunit interactions that regulate the portal monomer’s folding into the correct conformation for oligomerization into stable 12-mer rings.

**IMPORTANCE:**

To be infectious, most double-stranded DNA (dsDNA) viruses and bacteriophages package their DNA into the capsid via the dodecameric portal protein vertex. This first requires that portal monomers assemble into a portal ring that becomes incorporated into the capsid, but this oligomerization process is not well defined. Here, we show that mutating a highly conserved basic motif, _272_KRRR_275_, in the bacteriophage P22 portal protein wing domain impairs proper ring assembly and incorporation, despite not being involved in inter-subunit contacts. This defect arises from altered secondary structure of the portal monomer, emphasizing the importance of specific intra-subunit interactions necessary for portal oligomerization. These results further our understanding of essential protein-protein interactions involved in viral assembly and provide insight into key interactions that can aid the development of antiviral drugs targeting portal oligomerization and incorporation.

## INTRODUCTION

Many double-stranded (ds) DNA viruses and bacteriophages require the assembly of a precursor capsid (procapsid) (1–3) into which their genome is packaged through a unique vertex (4–7). Occupying this vertex is a homo-dodecameric ring structure known as the portal protein complex that initiates procapsid (PC) formation (8–10) and acts as the channel for DNA translocation into and out of the capsid during assembly and infection. The incorporation of only a single portal ring relies on interactions between portal monomers and scaffolding proteins (8–14). Despite the lack of sequence similarity across viral and bacteriophage (phage) portals, they share highly conserved structural features, enabling comparisons between them. The conserved “portal-fold” consists of up to five domains: the barrel (observed in phages P22 [15], Sf6 [16], and PaP3 [17]), crown, wing, stem, and clip (6). Each of these domains is critical for the portal’s interactions with different assembly proteins at distinct stages of maturation. The barrel and crown are sites for ejection protein interactions (18–20), the wing is involved in scaffolding protein binding when the portal is in its PC conformation (21–23), and the clip is a dual binding site for both the terminase complex (24, 25) and plug proteins (26, 27), during and at the cessation of DNA packaging, respectively. Given its essential role in infectious virus production and high degree of structural conservation across dsDNA viruses, the portal protein represents an attractive target for anti-viral drug development (28).

The assembly pathway of bacteriophage P22 has been extensively studied, providing a robust model for characterizing specific protein-protein interactions between the assembly proteins in more depth. Its morphogenesis begins with the interaction of scaffolding protein (gp8) and portal protein (gp1) monomers to induce the oligomerization of the portal ring that acts as the site of nucleation around which coat proteins (gp5) are assembled to form the PC (29). dsDNA is subsequently packaged into the procapsid until the capsid head is full by an ATP-dependent motor called the terminase (gp2 + gp3) complex (30). During maturation, the portal protein undergoes a conformational change from its PC conformation to the more compact mature virion (MV) conformation, which is characterized by the extension of the barrel domain (15). Though the exact step in the process is not known, DNA packaging is believed to initiate the conformational signal (15). As the signal propagates, the wing domain undergoes a dynamic movement allowing for the release of scaffolding protein from around the portal (15, 21), and the hammer loop of the wing domain rotates away from the crown domain, allowing the barrel to extend (15). Ultimately, these structural rearrangements of the portal to its MV conformation trigger the disassociation of the terminase complex (15, 31) and the recruitment of the plug proteins (32) and tail machinery to complete the mature virion. However, the specific protein-protein interactions between scaffolding and portal proteins necessary to assemble portal into the correct dodecameric ring have not been elucidated.

**Fig 1:**
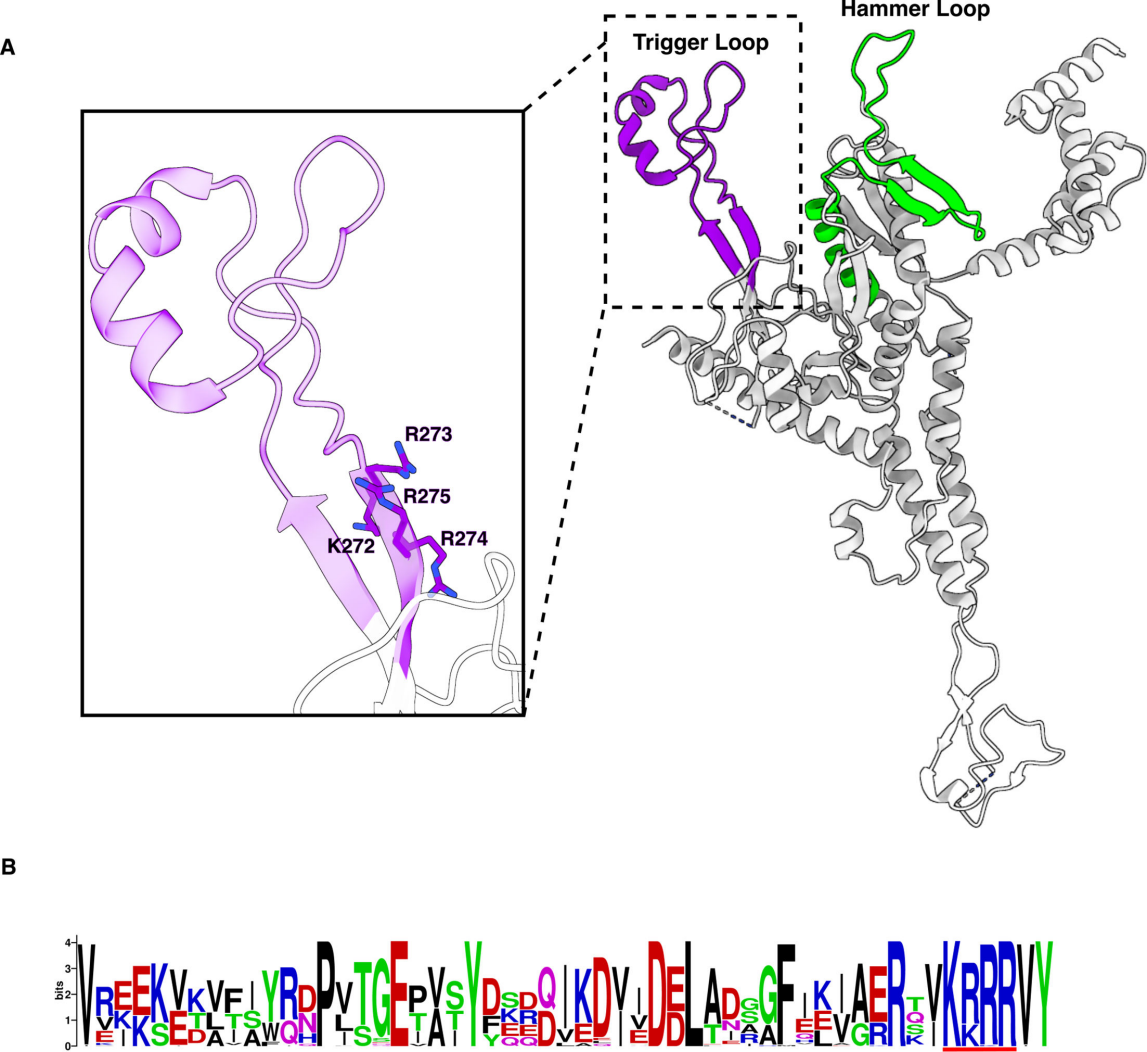
Location of the conserved _272_KRRR_275_ motif in the portal wing. (**A**) Structure of the procapsid portal protein monomer. The trigger loop is colored in purple, and the hammer loop in green. The amino acids comprising the _272_KRRR_275_ motif (colored by heteroatom) are located in one strand of a two-stranded anti-parallel β sheet. PDB: 9JGA. (**B**) The consensus sequence of the trigger loop (residues 226–277 in P22). The weblogo (34) was generated from a multiple sequence alignment of 65 P22-like phage portal protein sequences performed with ClustalOmega. The _272_KRRR_275_ motif is underlined in red.

In P22 (33) and other phages, the portal wing domain has been implicated as a binding site for the scaffolding protein. Structural evidence from phages φBB1 (22) and HK97 (23) suggests that the portal wing interacts with scaffolding protein or the analogous “delta domain” through electrostatic interactions. Within the wing domain of phage P22, there are two surface-exposed loops, termed trigger (residues 226–277) and hammer (residues 456–505) (Fig. 1A), that undergo significant conformational changes between the PC and MV states of the portal (15). The trigger loop contains a highly conserved stretch of positively charged amino acids (_272_KRRR_275_) (34) (Fig. 1B) that we hypothesize are crucial for interactions with the scaffolding protein that drive proper ring oligomerization and incorporation in phage P22.

In this study, a combination of *in vivo* and *in vitro* techniques is used to probe the effects of altering the highly conserved _272_KRRR_275_ motif on portal conformation, oligomerization, incorporation, and interaction with scaffolding protein. We find that this motif is necessary for incorporation of the portal into PCs, but rather than directly affecting interactions with scaffolding protein, our data show the wing domain is critical for stability and interaction between portal subunits.

## RESULTS

### The alanine substitutions to the _272_KRRR_275_ motif result in a dramatic decrease in phage production

**Fig 2:**
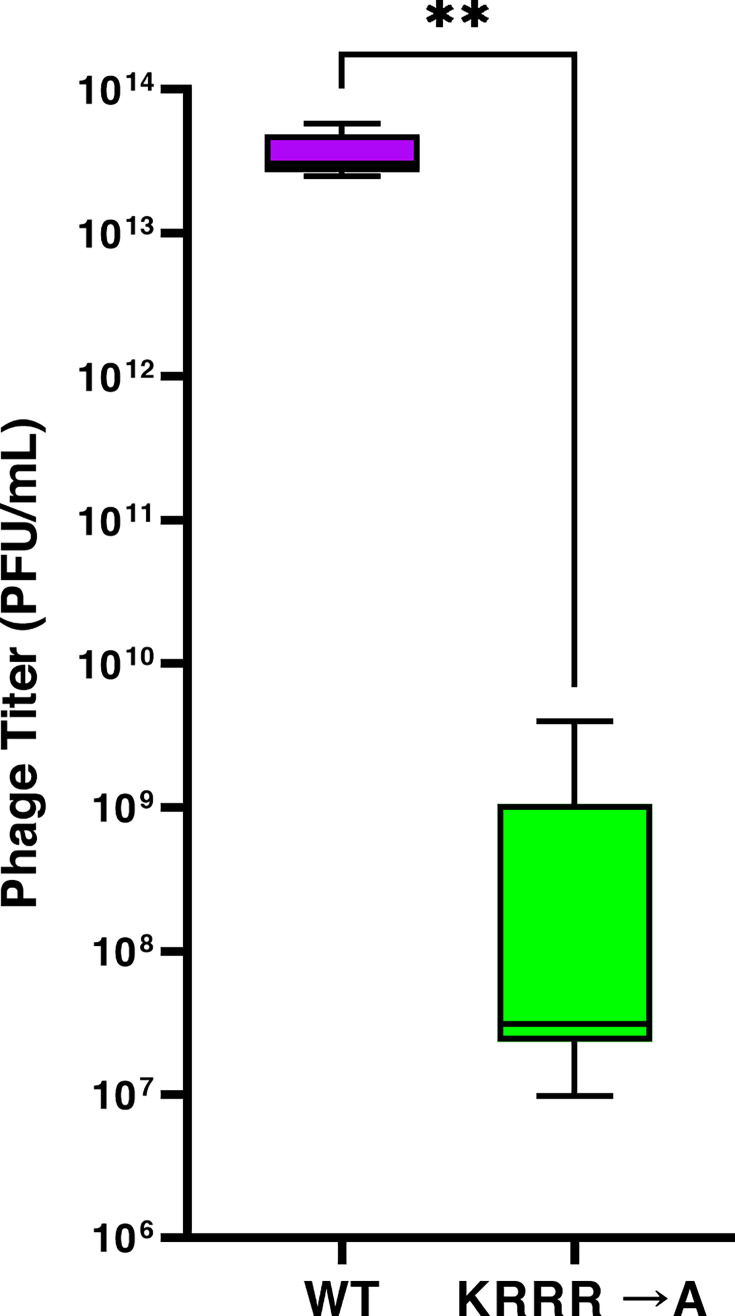
The KRRR→A mutation causes a reduction in phage production. Lysates from replicates of prophage induction of WT and KRRR→A were plated on the amber-suppressing strain *Salmonella* DB7155 to determine the phage titers, which are depicted as a box and whisker plot. The horizontal solid line indicates the mean of the data. Significance was determined by an unpaired *t*-test with Welch’s correction in GraphPad Prism. Asterisks (**) indicate a *P*-value less than or equal to 0.01. *P*-value = 0.0011.

Alanine substitutions (KRRR to AAAA, referred to as KRRR→A) were introduced into the P22 prophage UC-0937 (Table 1) by recombineering to assess the role of the conserved _272_KRRR_275_ motif in the portal wing (see Materials and Methods for details on strain construction). Wild-type (WT) and KRRR→A variant prophages were induced at 37°C with Carbadox (35), and the resulting phage titers were determined by plating on the *Salmonella* strain DB7155 (36). The quadruple substitution led to a 4–6 log decrease in the number of infectious phage particles produced (Fig. 2), suggesting a defect in either portal incorporation or portal functionality *in vivo*.

**TABLE 1:** Bacterial strains used in this study

Strain name	Genotype
UB-2235	*galK::TetRA*-1, ∆Fels-1, ∆Fels-2, ∆Gifsy-1, ∆Gifsy-2 (P22 UC-0937^*a*^)
MNL01	UB-2235 (P22 UC-0937 with *galK* replacing gp1 DNA sequence encompassing trigger loop, from bp 608 to 908)
MNL02	UB-2235 (P22 UC-0937 with KRRR to AAAA substitution, gp1 a.a. residues 272–275)

^*a*^ Phage UC-0937 is the “wild-type” parent of the portal variant made in this study. This phage has an *ImmI-sieA* deletion (37), an amber mutation in gene 13 to prevent host cell lysis, and a* c1-7 *clear plaque mutation (38).

### Procapsid and phage morphology are unaffected by the alanine substitutions

**Fig 3:**
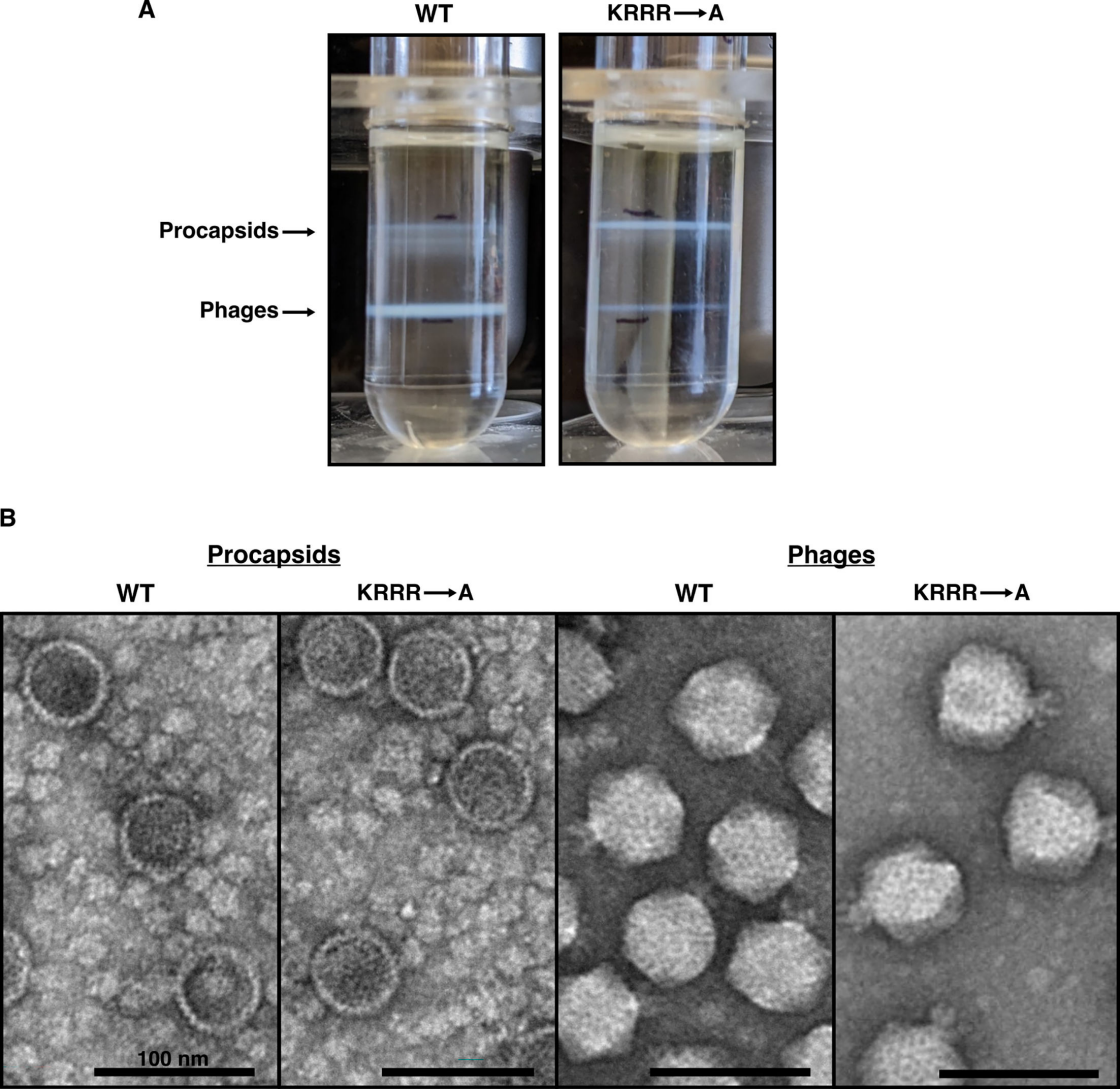
The yield, but not morphology, of procapsid and phage particles is affected by the KRRR→A variant. (**A**) Cesium chloride density gradients of lysates post-prophage induction. Fewer phages and more procapsids are produced by the portal variant prophage induction compared to WT, as observed by the size difference of the bands. (**B**) Negative stain transmission electron micrographs of particles purified by CsCl gradients in panel A. Both WT and the portal variant prophage inductions produce procapsids and phages with the correct morphologies.

Since PC assembly is initiated at the portal ring (29), one possibility for the reduction in viable phages observed for the KRRR→A variant is the formation of aberrant particles. To address this, WT and variant particles produced by induction of the prophages were purified using cesium chloride (CsCl) step gradients with a 25% sucrose layer on top. Phages sediment to the interface of the 1.4 and 1.6 g/cc CsCl steps, whereas PCs and any aberrant particles will sediment at the interface of the 25% sucrose and 1.4 g/cc CsCl step. The KRRR→A variant lysate showed a more prominent PC band and a diminished phage band compared to the WT lysogen lysate (Fig. 3A). This observation suggests that fewer PCs were able to mature into phages for the KRRR→A portal variant. Negative stain transmission electron microscopy of the CsCl-purified particles revealed no observable differences in morphology between WT and variant PCs or phages (Fig. 3B). There were also no observed defects with the phage heads, such as burst heads or abortive packaged phages leaking their DNA, indicating no issues with the DNA packaging signal of the variant portal. Since procapsid-like particles can assemble correctly in the absence of a portal (39), the PCs produced from the KRRR→A prophage induction may not contain a portal, thereby preventing many of the particles from maturing.

### Maturation of variant procapsids is inhibited

**Fig 4:**
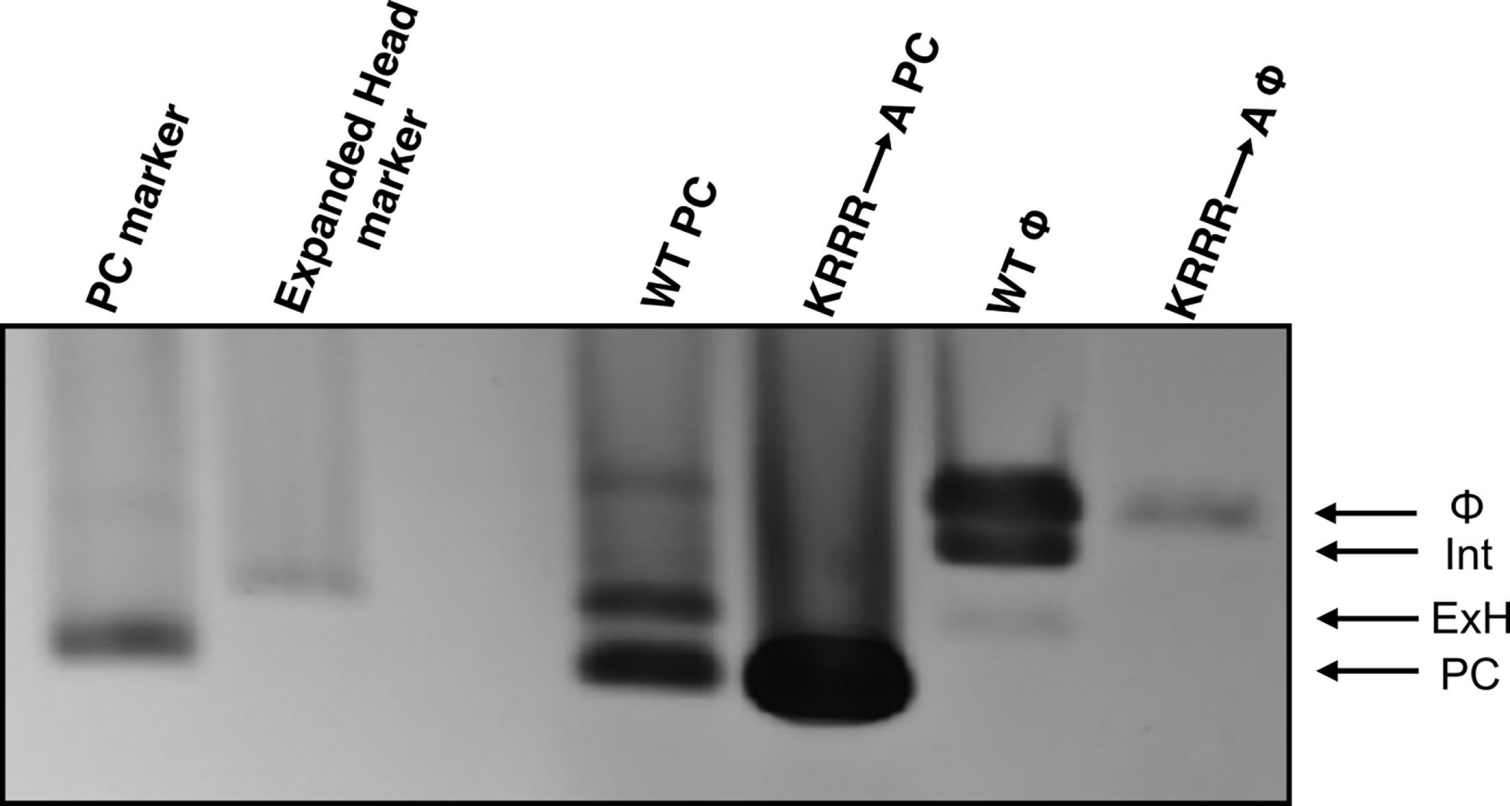
KRRR→A portal prevents the formation of maturation intermediates. CsCl-purified procapsid (PC) and phage (Φ) bands were run on a 1% native agarose gel and stained for protein with Coomassie Brilliant Blue. The WT PC has three bands corresponding to PC, expanded head (ExH), and Φ, while the KRRR→A variant PC has a single band corresponding to PC. The WT Φ has two bands corresponding to an intermediate (Int) and Φ, while the KRRR→A variant has a single Φ band.

By native agarose gel electrophoresis, WT P22 has been shown to have particles that adopt two intermediate maturation states between procapsids and phages (40). One has an electrophoretic mobility that is slower than procapsids on a native agarose gel and has been identified as an expanded head state, which is likely a result of abortive DNA packaging. The other has a faster mobility than phages. Its maturation state has not been described, but it is likely a packaging intermediate. In Fig. 4, a similar electrophoretic pattern for the WT particles from prophage induction is seen, where the PCs purified by CsCl gradients show a band corresponding to PCs, as well as the secondary expanded head band on a native agarose gel. For the CsCl-purified WT phages, a phage band, as well as that of the uncharacterized intermediate, can be observed (40). For the KRRR→A variant particles, in contrast, there is a single PC band and a single phage band without the presence of the expanded head or uncharacterized phage intermediates, suggesting that the majority of the KRRR→A variant particles are not efficient at DNA packaging and may not have all the head proteins required for packaging and maturation.

### Portal incorporation is reduced in procapsids with the variant portal protein

**Fig 5:**
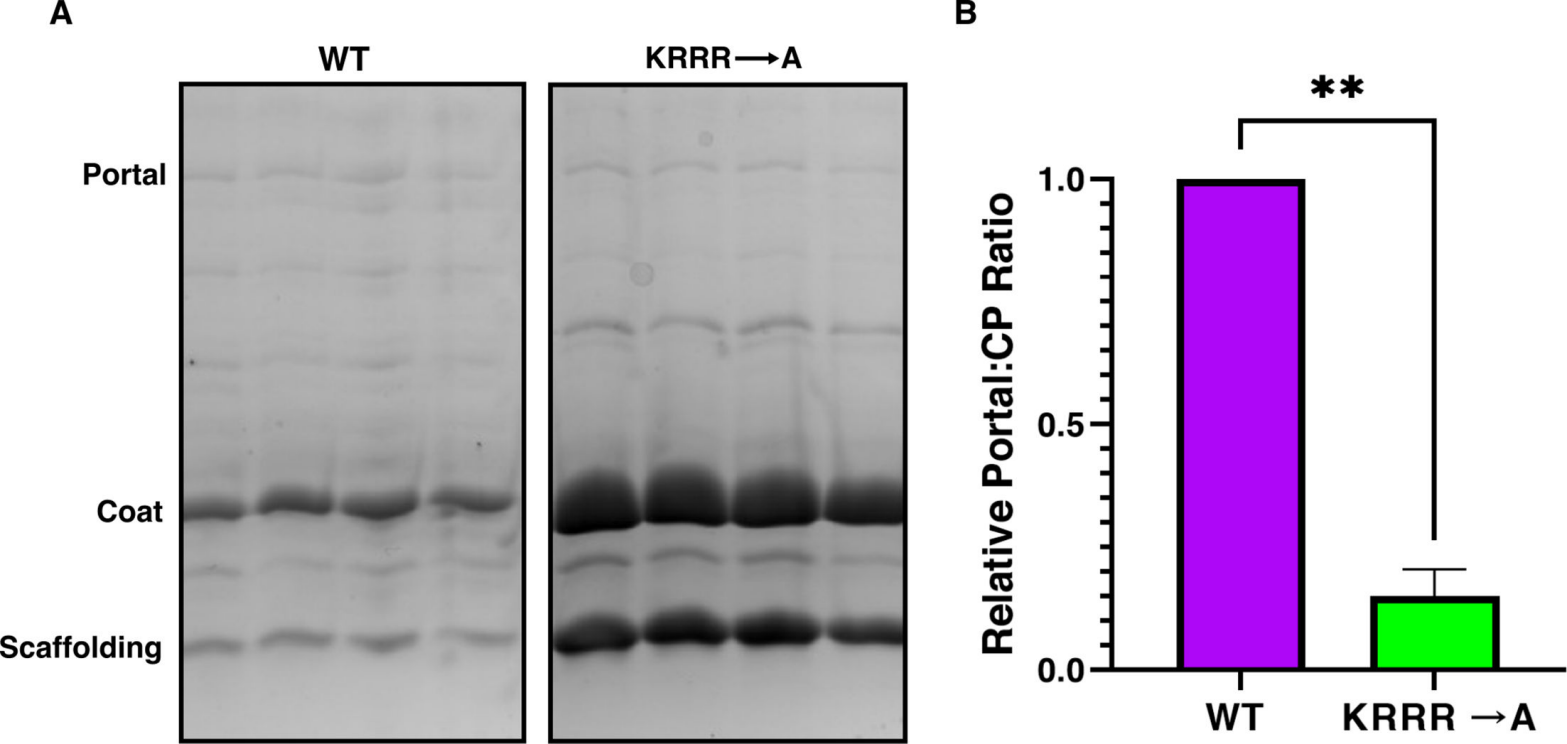
Quantification of portal incorporation. (**A**) SDS-PAGE analysis of peak procapsid fractions from sucrose gradients of WT and variant. (**B**) The relative portal:coat protein ratio for WT vs variant determined by densitometry of the gels in panel A. Significance was determined by an unpaired *t*-test with Welch’s correction in GraphPad Prism. Asterisks (**) indicate a *P*-value less than or equal to 0.01. *P*-value = 0.0014.

The protein composition of the PCs was analyzed to determine if they contained the correct ratios of assembly proteins needed for maturation. To examine whether the KRRR→A substitution affects portal incorporation into PCs, WT and variant lysates from prophage inductions were applied to 5%–20% sucrose gradients and the fractions analyzed by SDS-PAGE. Several observations can be made from the gels (Fig. 5). First, despite equal input volumes of lysate from prophage induction applied to the sucrose gradient, the WT variant particles showed less protein in the peak PC fractions compared to those from the KRRR→A prophage induction (Fig. 5A), consistent with the CsCl gradient data (Fig. 3A). Second, a decrease in the amount of portal in the peak PC fractions for the KRRR→A variant compared to WT was observed (Fig. 5A). This decrease was quantified by densitometry of the portal and coat protein bands. The intensities of these bands were used to calculate the KRRR→A variant portal:coat protein ratio relative to the WT portal:coat protein ratio, which was set to 1. For the variant, the ratio was reduced to 0.15 (Fig. 5B). The decrease in portal incorporation could explain the decrease in phage production and the cessation of assembly at the PC stage, since a portal is required for maturation. This portal incorporation defect suggests a potential disruption in portal-scaffolding protein interactions, which could affect ring oligomerization and/or subsequent ring incorporation.

### The KRRR→A substitution causes a portal oligomerization defect *in vitro*

In the absence of scaffolding protein, portal rings purified from plasmid expression systems for phages, such as T7 (41), T3 (42), SPP1 (43), and herpes simplex virus type 1 (44), can form oligomeric assemblies ranging from 11 to 14-mers, though *in vivo* only 12-mer portal rings are incorporated into procapsids. To determine if the P22 variant portal monomers can assemble into the correct dodecameric rings *in vitro*, WT and variant portal monomers were purified by metal affinity chromatography, subsequently concentrated to force ring assembly, and further purified by size exclusion chromatography (SEC) (45). From the SEC chromatograms, the KRRR→A rings eluted from the column earlier than WT, at an elution volume of ~12.5 mL compared to ~13.5 mL, suggesting the variant rings are larger than WT rings (data not shown).

**Fig 6:**
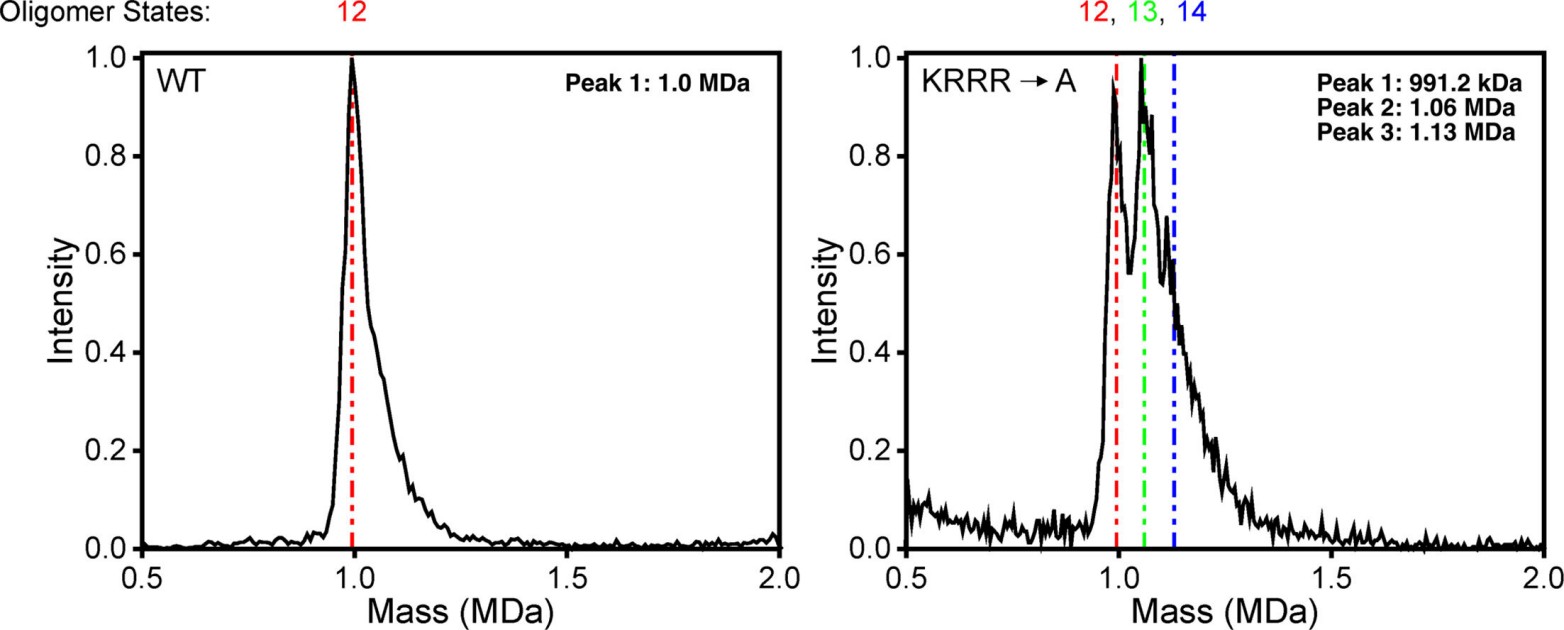
Charge detection mass spectrometry of portal rings reveals variable oligomeric states for the KRRR→A variant. The CD-MS spectra for WT and variant *in vitro* assembled portal rings are shown, with the observed data in black and colored vertical lines indicating peak positions and their corresponding oligomeric states. The expected mass of the portal monomer is ~82.7 kDa, the 12-mer is 993 kDa, the 13-mer is 1.075 MDa, and the 14-mer is 1.158 MDa.

Charge detection mass spectrometry (CD-MS) was employed to more accurately determine the oligomeric state of the rings. This method is a native, single-particle technique that can measure the mass of large protein complexes (46). CD-MS was used to find the oligomeric state of mutant portal rings (45) and show that WT P22 portal monomers assemble exclusively into 12-mer rings (8). Here, the data for WT rings were replicated, showing a single peak centered ~1 MDa in the mass spectrum corresponding to the mass of a 12-mer ring (Fig. 6). While this is larger than the theoretical mass of 993 kDa, measured masses for large molecular assemblies can be ~1% greater because of incomplete removal of bound buffer or salt molecules (47). On the other hand, there are three peaks in the mass spectrum for the KRRR→A mutant corresponding to 12, 13, and 14-mer portal rings (Fig. 6). The CD-MS data indicate that in the absence of scaffolding protein, alanine substitutions to the _272_KRRR_275_ motif induce improper ring assembly, which could explain the lack of portal incorporation *in vivo*.

**Fig 7:**
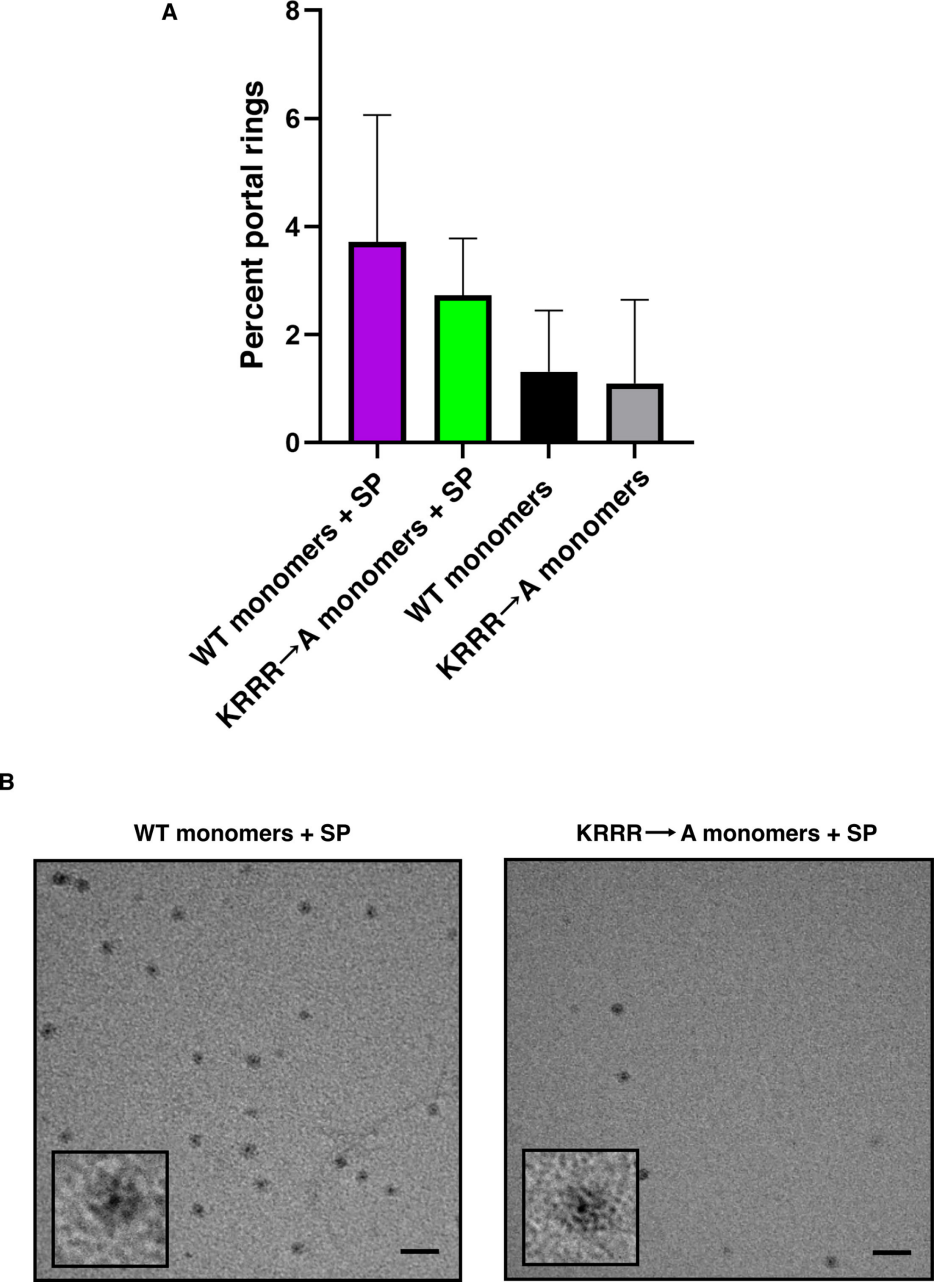
Ability of scaffolding protein to induce ring formation *in vitro*. (**A**) Densitometry was used to quantify the percentage of portal rings produced from *in vitro* oligomerization assays of WT and variant monomers with and without scaffolding protein (SP). The portal protein band intensities were summed across the entirety of the 10% SDS-PAGE gel, and the intensities of portal in the ring-containing fractions were normalized to the total portal protein intensity. The normalized values were summed and expressed as percent portal rings. Statistical significance was determined using a one-way ANOVA in GraphPad Prism. *N* = 3 for oligomerization reactions with WT monomers, WT monomers + SP, and variant monomers + SP. *N* = 2 for variant monomers. (**B**) Negative stain transmission electron microscopy of one portal ring fraction taken at 150,000× magnification. The scale bar is 50 nm.

The ability of scaffolding protein to trigger portal protein oligomerization was determined by incubating WT and variant portal monomers with scaffolding protein for 4 h at room temperature in a HEPES potassium acetate (KAc) buffer, and the reactions were applied to sucrose gradients (8). Fractions were run on 10% SDS-PAGE gels, and the portal protein band intensity was quantified. The intensities of the portal ring fractions were normalized to the total portal protein in the reaction and expressed as a percentage. As shown in Fig. 7A, the variant produced a slightly lower percentage of rings than WT, though this difference was not statistically significant. Thus, scaffolding-induced ring assembly is not impaired by the portal variant.

Further analysis of portal rings from the WT and variant gradients by negative stain transmission electron microscopy (TEM) showed that the variant rings assembled by scaffolding protein exhibit a normal morphology (Fig. 7B). The rings do not appear to be malformed or aggregated when compared to the scaffolding protein-induced WT rings, though with negative stain TEM it is not possible to distinguish between 12-mer vs 13- or 14-mer rings. While rings are still produced, they may exhibit structural changes that cannot be observed by TEM, which diminish their incorporation *in vivo*.

### The secondary structure of portal is altered by the substitutions

**Fig 8:**
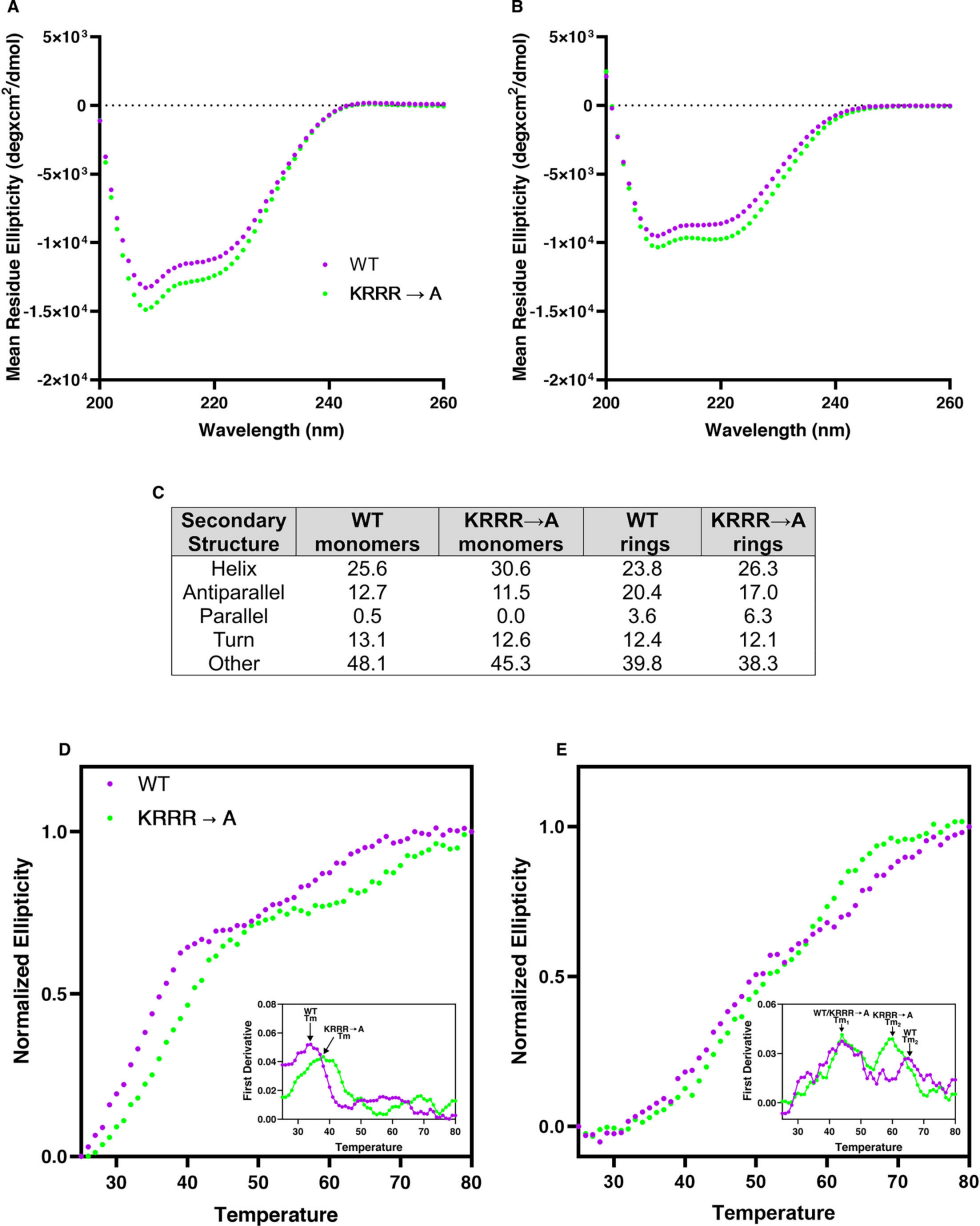
The secondary structure of portal monomers and rings is altered by substitutions to the _272_KRRR_275_ motif. (**A and B**) The averaged CD spectra of WT and variant portal monomers (**A**) and rings (**B**) collected at 20°C. Three individual protein preparations were used to obtain the average. (**C**) The predicted secondary structure derived from the spectra of monomers and rings in panels A and B using BeStSel. (**D and E**) Thermal stability of purified WT and variant portal monomers (**D**) and rings (**E**) measured by CD at 222 nm from 25°C to 80°C. Two individual protein preparations were used to acquire the average. The melting curves were smoothed in OriginPro using the Savitzky-Golay method with a polynomial order of 2 and a window size of 5. The first derivative of the smoothed data was acquired and plotted vs temperature (insets of D and E) to determine the melting temperatures (Tm).

The ring oligomerization defects described above could be due to structural differences between the WT and KRRR→A portal monomers. Circular dichroism (CD) spectroscopy was used to evaluate the secondary structure of purified WT and KRRR→A portal monomers and rings to determine whether the substitutions affect the fold of the portal protein. Both WT and KRRR→A monomer spectra shared characteristics of a highly α-helical protein, observed by the negative ellipticity at 208 and 222 nm (Fig. 8A). The spectrum of KRRR→A reproducibly showed a slight increase in α-helicity at 222 nm compared to WT monomers. Consistent with the secondary structural differences of the monomers, the KRRR→A rings also exhibited an increase in α-helical content at 222 nm (Fig. 8B). The percentage of secondary structural elements was predicted using BeStSel (48). The KRRR→A monomers were predicted to have ~30% helical content in comparison to the WT monomers, which have ~25% (Fig. 8C). A small (2.5%) increase in α-helicity was also observed for the KRRR→A rings. Additionally, both variant monomers and rings displayed a slight reduction in anti-parallel β-sheet secondary structure.

Next, the thermal stability of the monomers and rings was investigated. The thermal melts of the monomeric proteins indicate a cooperative unfolding transition between ~30°C and 45°C (Fig. 8D). The melting temperature (Tm) of the WT monomer is ~34°C and the Tm of the variant monomer is ~38°C for this first transition, as determined by the plot of the first derivative of the Savitzky-Golay smoothed data (49) (Fig. 8D, inset). There is a broad and non-cooperative second transition for each protein, though the variant’s is shifted to a higher temperature. Both transitions suggest an increase in stability for the KRRR→A variant. The thermal denaturation curve for the portal rings exhibited two melting transitions (45, 50). The first transition occurs ~35°C–51°C, and the second from ~55°C to 67°C (Fig. 8E). For WT, these transitions have Tm’s of 44°C and 65°C, respectively. Variant rings exhibit an equal Tm of 44°C for the first transition but have a lower Tm of 60°C for the second transition (Fig. 8E), suggesting the KRRR→A substitutions also influence the stability of portal rings.

### The KRRR to alanine substitutions elicit a change in tertiary structure of the rings

Changes in the tertiary structure of the variant portal rings were examined by limited proteolysis with α-chymotrypsin. This protease cleaves on the C-terminal side of aromatic amino acids (Trp, Tyr, Phe). Therefore, its cleavage activity serves as a useful probe for detecting conformational differences by targeting exposed hydrophobic patches.

**Fig 9:**
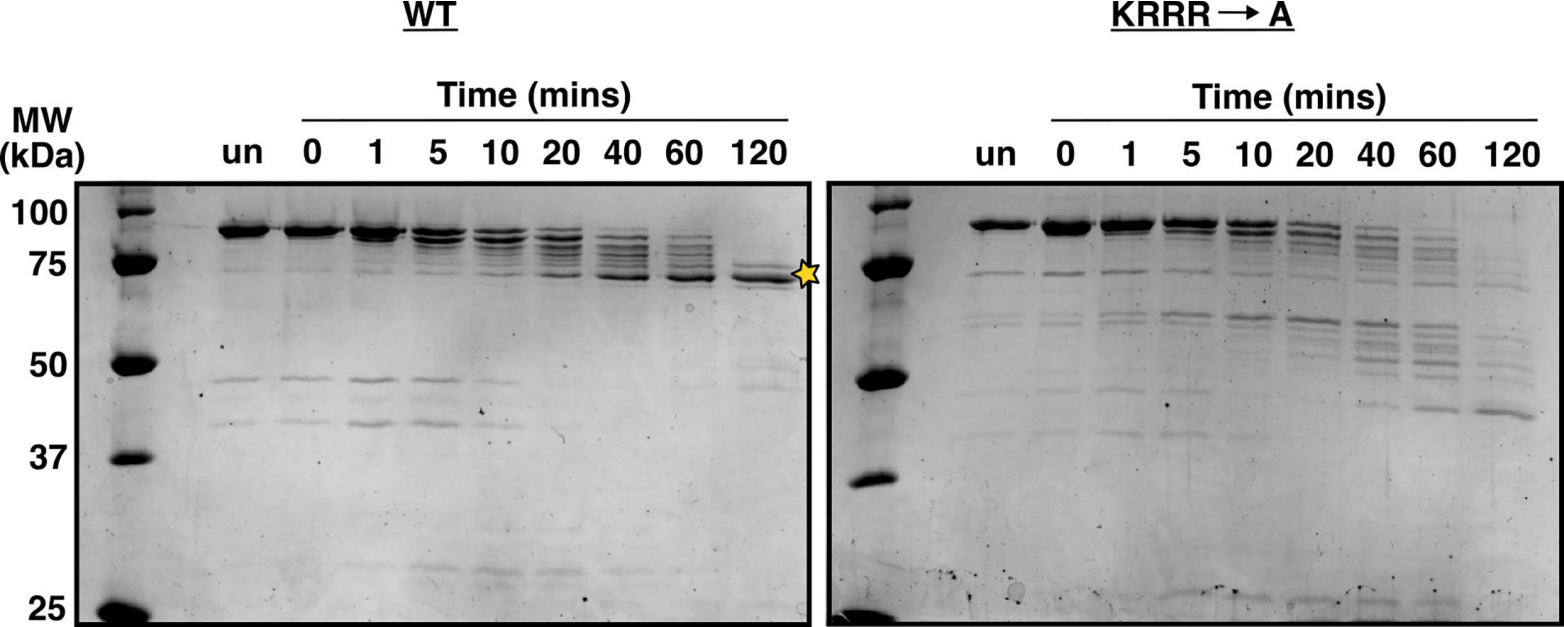
Tertiary structure of the ring is altered by the substitutions. Limited proteolysis of portal WT (left) and variant (right) portal rings by α-chymotrypsin at 37°C. Undigested (un) and time course samples were run on a 10% SDS-PAGE gel. The yellow star in the WT gel indicates the protein band that was cut out from the gel and analyzed by mass spectrometry following in-gel digestion with trypsin.

WT portal rings are slowly digested to a major product of ~75 kDa (Fig. 9). Mass spectrometry analysis of this protease-resistant fragment revealed that it contains peptides of the core of the portal, including the N-terminus (amino acid residues 2–624), but lacks peptide fragments from the last ~100 amino acid residues corresponding to the C-terminal barrel (data not shown). In contrast, the KRRR→A portal rings are completely digested, with no stable fragments present. These results indicate that the tertiary structure of the variant portal is altered, thereby increasing the accessibility of chymotrypsin cleavage sites in the core that are normally protected from digestion in WT portal rings.

## DISCUSSION

dsDNA viral assembly is a coordinated process carried out by the protein-protein interactions of several viral proteins. An essential protein in this process is the portal complex, whose high structural dynamism allows it to adopt a multitude of conformations to interact with assorted viral proteins at different stages of assembly. The first interaction partner of the portal is the scaffolding protein, which drives portal ring oligomerization to form the assembly nucleation complex (8). Here, we investigated if the conserved _272_KRRR_275_ motif in the portal wing domain is a scaffolding protein binding site.

### The KRRR motif is not a binding site for scaffolding protein

**Fig 10:**
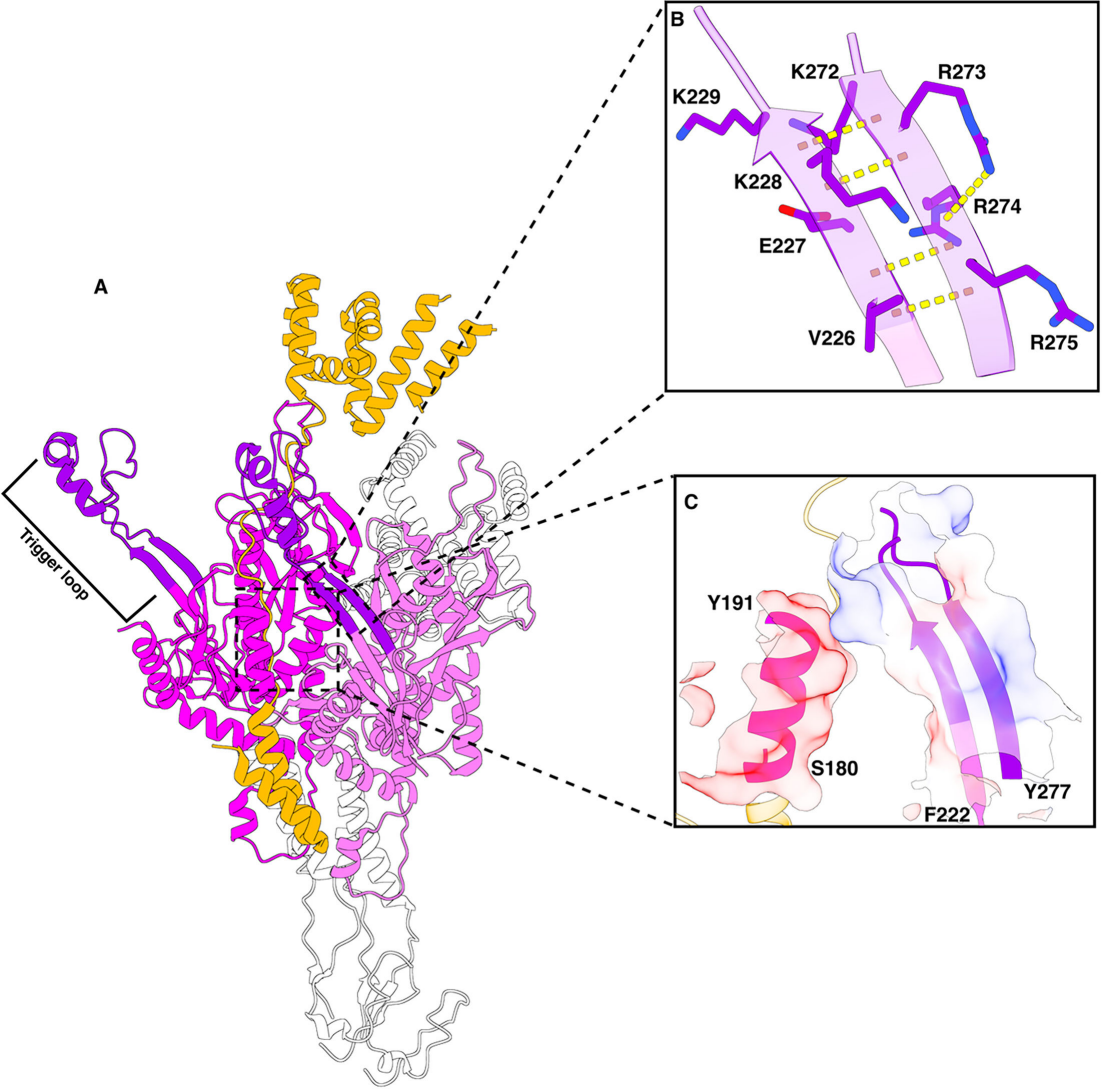
***I****n situ* procapsid structure of phage P22 portal and scaffolding protein. (**A**) Two portal monomers whose wing domains (colored pink and purple) form a cleft for scaffolding protein (colored in orange). PDB: 9KYW. (**B**) Hydrogen bonds (colored in yellow) between the _272_KRRR_275_ motif and residues 226–229 on the opposing β-strand stabilize the β-sheet. (**C**) Electrostatic interactions between the β-sheet and α-helix of adjacent monomers.

We hypothesized that the conserved _272_KRRR_275_ motif could be a scaffolding protein binding site based on structural evidence from P22 (33) and other phages (22, 23). However, our results indicate that the scaffolding protein does not directly bind to these residues via electrostatic interactions, but rather this motif is important for establishing the proper portal conformation necessary for ring oligomerization and incorporation into the PC. In earlier cryo-EM structures (PDB ID: 8PHP and EMDB ID: EMD: 29391) (22, 23), the scaffolding protein is shown to interact with the portal wing domain in a similar region to where the motif is located. A recent cryo-EM structure of the P22 procapsid has revealed the structure of the portal in complex with scaffolding protein *in situ* (PDB: 9KYW) (21). In this structure, a majority of the scaffolding protein is observed interacting in between a cleft formed between two adjacent portal protein monomer wing domains (21) (Fig. 10A) rather than the exterior trigger loop of a single monomer. In addition, the structure shows that the portal-scaffolding protein interaction is predominantly driven by hydrophobic rather than electrostatic interactions. A second region of direct portal-scaffolding interaction was found to be the crown domain (residues 495–504) (21). The data presented here are consistent with this recent structure. Despite not being a direct binding site for scaffolding protein, substituting the _272_KRRR_275_ motif with alanines has dramatic effects on portal assembly and incorporation *in vivo* and *in vitro*.

### The _272_KRRR_275_ motif is necessary for proper ring oligomerization

Previous work using heterologously expressed WT P22 portal monomers showed they will assemble into 11-mers and 12-mers (ratio of 70:30) (51, 52) or exclusively 12-mers depending on the purification procedure (53, 54), but never any larger assemblies. Here, using the purification method that produces exclusively 12-mer WT rings, the KRRR→A monomers assembled into 12-, 13-, and 14-mer portal rings, demonstrating a defect in oligomerization (Fig. 6).

While the portal wing domain is a site of subunit-subunit interactions (9, 55, 56), the _272_KRRR_275_ motif is not at a subunit interface (Fig. 1), which might be expected for a region that affects oligomerization. However, previous work has shown that substitutions to wing domain residues that are not at a subunit interface can affect portal assembly. Substitution of residue W211 to alanine results in the formation of hyperstable rings (45), while substitution of C283 to serine causes hyperstable monomers that are resistant to ring formation (57). Residue C283 resides just after the _272_KRRR_275_ motif, suggesting that this region of the wing is important for intra-subunit interactions that drive the correct folding of the portal monomer into the conformation required for proper dodecameric ring assembly.

### What is impeding portal incorporation *in vivo*?

Despite changes in secondary structure and thermal stability observed by CD of the KRRR→A portal monomers, they are still capable of being assembled into rings by scaffolding protein *in vitro*, suggesting that the substitutions do not impair the scaffolding protein binding site (Fig. 7). However, the rings that are assembled in the presence of scaffolding protein both *in vitro* and *in vivo* may share the unique attributes of the self-assembled variant rings resulting in incompetence for incorporation into PCs. Notably, the rings may be of the incorrect oligomeric state (Fig. 6) and have altered secondary and tertiary structures (Fig. 8 and 9). Previous investigation of WT portal rings by CD thermal melts revealed the same two melting transitions observed in this study; however, the unfolding transitions were not attributed to particular domains (50). Here, we see that substitutions to the wing domain cause a decrease in the Tm of the second transition (Fig. 8E), indicating that this transition is likely related to unfolding of the wing. Thus, destabilization of the wing domain does not affect scaffolding protein binding but rather causes differences in its organization or orientation around the variant rings.

In the *in situ* structure of the portal ring and scaffolding protein (21) (Fig. 10A), the _272_KRRR_275_ motif folds into one strand of a two-stranded β-sheet that is stabilized by hydrogen bonds between the KRRR residues and the adjacent strand (Fig. 10B). This β-sheet is part of a larger loop that reaches across (counterclockwise around the ring) the cleft made by adjacent portal monomers, “locking” the scaffolding protein in place (Fig. 10A). By substituting the _272_KRRR_275_ motif with alanines, this secondary structural feature is likely destabilized. Although the _272_KRRR_275_ motif is not at a subunit–subunit interface, the β-sheet may make electrostatic interactions with a helix (residues S180-Y191) of the adjacent monomer (Fig. 10C). Disruption of the interactions between the regions that cover the cleft may affect the portal-scaffolding protein interaction by altering how the scaffolding protein is situated in the cleft. This then could influence the downstream interactions of the portal:scaffolding complex with coat protein, preventing its incorporation into PCs. In addition, substitutions to the _272_KRRR_275_ motif may indirectly affect the inter-subunit electrostatic interactions between monomers, leading to the formation of rings having the incorrect oligomeric state.

### A positively charged motif appears exclusive to large podoviridae portals

The KRRR motif is not present in the portal proteins of the siphophage SPP1 or the myophage T4, suggesting that this motif may be restricted to podophages. However, a similar charged motif is also absent from the portals of phages T7 and Φ29, both podoviruses. What differentiates these portals from those of P22 is their size. The P22 portal is much larger at 725 amino acids compared to 536 and 309 for T7 and Φ29, respectively. Additionally, P22 has a barrel domain that is not present in these smaller portals. Therefore, we examined two larger podovirus portals from phages Sf6 and Pa223 for the motif.

**Fig 11:**
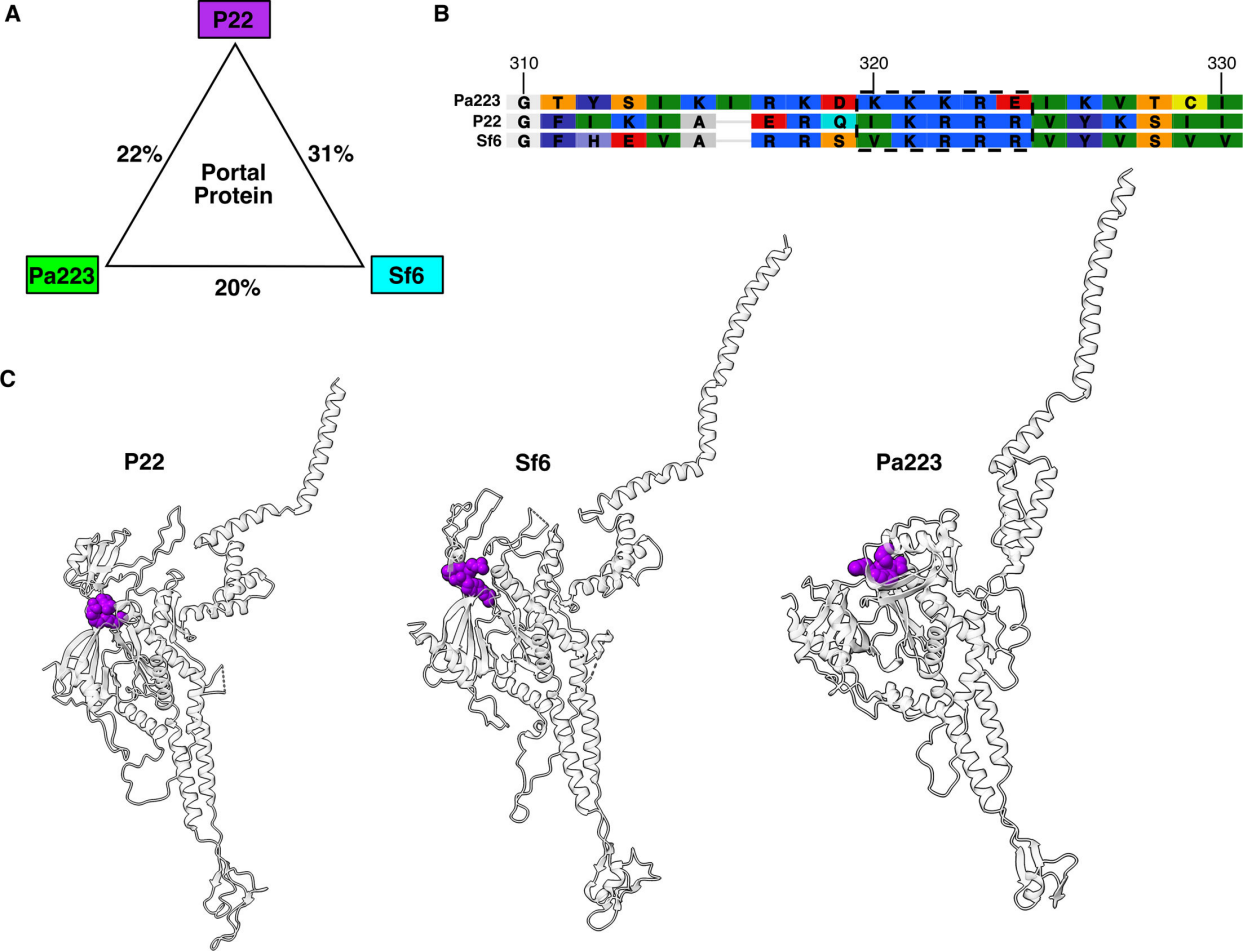
Conservation of the positively charged motif. (**A**) Percent sequence identity between phage P22, Sf6, and Pa223 portals. Percent identity was determined using Clustal Omega following a multiple sequence alignment. (**B**) Multiple sequence alignment between P22, Sf6, and Pa223 from residue 310 to 330, which contains the _272_KRRR_275_ motif, is outlined in the dashed box. The NCBI multiple sequence alignment viewer was used for visualization with RasMol amino acid coloring. (**C**) Structures of P22 (PDB: 9JG6 [21]), Sf6 (PDB: 7SFS [16]), and Pa223 (PDB: 9NWM [58]) portal monomers with the positively charged motif in the wing domain depicted as purple spheres. The motif is residues 272–275 in P22, 280–283 in Sf6, and 190–193 in Pa223.

*Shigella flexneri* phage Sf6 and *Pseudomonas* phage Pa223 portals have low overall sequence identity to each other and P22 (Fig. 11A), but display a high conservation of positively charged residues in their wing domains (Fig. 11B and C). Despite the lack of overall sequence identity, the Sf6 portal retains the KRRR motif entirely (Fig. 11B), likely because of its close evolutionary relationship to P22. Notably, the presence of a positively charged motif in the wing domain is not exclusive to P22-like phages. Pa223, a member of the *Bruynoghevirus* genus (58), contains a KKKR motif in its portal located in a structurally similar position in its wing domain compared to P22 and Sf6 (Fig. 11B and C). These observations suggest that a positively charged motif in the wing domain is a conserved feature of large, barrel-containing portals, where it likely functions to maintain the correct portal fold and conformational changes during maturation. Smaller portals, such as those of T7 and Φ29, may not experience the same structural constraints and therefore may not need such a motif to achieve proper folding and oligomerization.

## MATERIALS AND METHODS

### Generation of portal protein mutants by recombineering

The wild-type (WT) P22 lysogen strain UB-2235 (genotype is given in Table 1), a generous gift from Dr. Sherwood Casjens, was used as the basis for recombineering (59, 60). The construction and use of this strain have been previously published (37, 61). In this work, the following strains were generated (Table 1). First, a *galK*-containing strain (MNL01) was produced by replacing the gene 1 (codes for the portal protein) target DNA sequence (codons for residues N203–P302) with a galK expression cassette that includes the *galK* gene and *em7* promoter. The *galK* gene was PCR amplified using *galK*-specific primers with 50 bp of gene 1 homology arms. Homologous recombination was facilitated by plasmid pKD46, which contains the lambda red recombination genes (62). Prophages containing *galK* were selected on M9 minimal media plates supplemented with galactose as the sole carbon source. To generate the prophage with the KRRR (aagcgtcgccgg) to AAAA (gcggcggcggcg) substitutions (MNL02), a dsDNA oligo was purchased from IDT with the desired substitutions and used as the *galK* replacement DNA. Recombinants were selected on M9 minimal media plates supplemented with 2-deoxygalactose. The substitutions were confirmed by Sanger sequencing. Before prophage induction, the pKD46 plasmid was removed by growing the lysogen at 37°C.

### Prophage induction

Cultures of the WT or portal mutant lysogen strains were grown overnight at 30°C in LB in the presence of chloramphenicol to maintain the prophage. A 300 mL culture of LB was inoculated with the overnight culture and grown to an OD_600_ of ~0.4 (~2 × 10^8^ cells/mL). The culture was induced with 1.5 µg/mL Carbadox (35) and grown for an additional 4 h at 37°C. Cells were harvested by centrifugation at 4,000 × *g* for 20 min, and the pellet was resuspended in dilution fluid (20 mM Tris, 100 mM MgCl_2_, pH 7.6). The cell suspension was lysed with chloroform in the presence of 100 µg/mL of DNase I. Cell debris and chloroform were pelleted by centrifugation at 4,000 × *g* for 20 min. The resulting supernatant, containing the phages, was collected and stored at 4°C.

### Determining phage yield

The lysate from prophage induction was titered on the amber-suppressing strain *Salmonella enterica* serovar Typhimurium, DB7155 (leuA414-am, hisC525-am sup^0^) (36). DB7155 cells were grown to mid-log phase at 30°C, centrifuged at 4,000 × *g* for 10 min at 4°C, and resuspended in 1/10th of the original volume of LB. Phage lysates were serially diluted in dilution fluid (20 mM Tris, 100 mM MgCl_2_, pH 7.6). One hundred microliters of cells and 100 µL of each lysate serial dilution were added to 2.5 mL of soft agar (0.8% wt/vol nutrient broth, 0.5% wt/vol sodium chloride, 6.5% wt/vol agar), mixed, and plated on LB agar (Lennox L agar, InVitrogen) plates. Plates were incubated at 30°C, and the resulting plaques were counted to calculate the phage yield.

### Cesium chloride gradient purification

Cesium chloride (CsCl) density step gradients were used to separate procapsids from phages. Gradients were prepared by layering 1.6 and 1.4 g/cc CsCl solutions prepared with dilution fluid, followed by 25% (wt/vol) sucrose, and topped with phage lysate. Samples were centrifuged at 70,000 × *g* for 1 h at 18°C. The procapsid and phage bands were extracted from the side of the tube with a needle, dialyzed against dilution fluid to remove residual CsCl, and then concentrated by centrifugation at 40,000 × *g* for 15 min at 4°C. Pellets were resuspended in 100 µL of dilution fluid.

### Agarose gel electrophoresis of CsCl gradient-purified particles

Procapsid and phage particles purified by a CsCl gradient were run on a native 1% SeaKem LE agarose gel in 1× TAM buffer (40 mM Tris base, 20 mM acetate, 20 mM MgCl_2_) for 90 min at 100 V. The gel was stained with Coomassie Brilliant Blue to visualize the proteins.

### Assessing particle morphology by negative stain transmission electron microscopy

The concentrated phage and procapsid samples from the CsCl gradients were diluted 1:4 in dilution fluid. Three microliters were adsorbed onto a 300-mesh carbon-coated copper grid (Electron Microscopy Sciences, Hatfield, PA) and stained with 1% uranyl acetate. Images were taken at 98,000× magnification on an FEI Tecnai G2 Spirit BioTWIN TEM.

### Sucrose density gradient analysis of *in vivo*-generated particles

Lysates (100 µL) from the induction of WT and portal variant lysogens were layered onto a linear 5%–20% sucrose gradient prepared with dilution fluid and centrifuged at 105,000 × *g* for 32 min. Fractions (100 µL) were collected from the top of the gradient using a positive displacement pipette and analyzed by SDS-PAGE.

### Generation of His_6_-tagged portal protein variant

Gene 1 with the codons for residues _272_KRRR_275_ changed to the codons for _272_AAAA_275_ was synthesized and cloned into the pET21b plasmid by Genscript. The plasmid was verified by DNA sequencing and transformed into *Escherichia coli* BL21 (DE3) cells for expression.

### Purification of portal protein rings and monomers

The expression and purification of the P22 portal protein have been described previously (15, 29, 45, 53). Briefly, cultures of BL21 (DE3) cells expressing His_6_-tagged variant and WT portal proteins were grown to an OD_600_ of ~0.6–0.7 at 30°C, followed by induction with 1 mM IPTG for 4 h at 28°C. The clarified cell lysate was applied to a 15 mL nickel column (Qiagen, Hilden, Germany), and the eluate was dialyzed overnight against portal buffer (20 mM HEPES (pH 7.5), 70 mM NaCl, 3 mM β-mercaptoethanol, and 1 mM EDTA). To prepare portal monomers (PMs), the protein was concentrated to less than 10 mg/mL using a Millipore-Amicon centrifugal filter with a 30 kDa molecular weight cutoff. Concentrated PMs were centrifuged at 23,000 × *g* in a Sorvall S120-AT2 rotor for 15 min at 4°C to remove any aggregates. To prepare portal rings, the protein was concentrated to ~50 mg/mL, as above, and incubated for 48 h at room temperature to promote oligomerization. The sample was centrifuged at 100,000 × *g* for 35 min at 4°C in a Sorvall S120-AT2 rotor to remove aggregates. Both PMs and rings were further purified by size exclusion chromatography using a Superose 6 Increase gel filtration column GL 10/300 (GE Healthcare) at a flow rate of 0.25 mL/min. Fractions were run on a NativePAGE 4%–16% bis-Tris gel (InVitrogen, Waltham, MA, USA), and those containing either PMs or portal rings were pooled and concentrated, as above.

### Charge detection mass spectrometry to determine ring oligomeric state

CD-MS measurements were performed by simultaneously measuring the mass-to-charge ratio (m/z) and charge of individual ions using a CD-MS instrument equipped with an electrostatic linear ion trap and embedded charge detection cylinder (Megadalton Solutions, Inc.). Experiments were conducted at an ion energy of 130 eV/z with a trapping time of 104.6 ms, resulting in a charge uncertainty of approximately 0.8 elementary charges. Samples were ionized and introduced into the CD-MS instrument via nano-electrospray ionization using a Triversa Nanomate (Advion Interchim Scientific, Ithaca, NY, USA). Samples of portal rings were buffer exchanged into 100 mM ammonium acetate (ThermoFisher) and 0.01% F68 (ThermoFisher) to help with stability and the buffer exchange process, using a Zeba protein desalting column (ThermoFisher) prior to the electrospray.

### Purification of scaffolding protein

To purify scaffolding protein without an affinity tag, 2 L of LB broth were inoculated with an overnight culture of T7 express cells transformed with plasmid pCMB162-A. This plasmid expresses only the scaffolding protein gene under the control of the T7 promoter. The cells were grown to an OD of ~0.7 at 37°C, followed by induction with 1 mM IPTG for 4 h and harvested by centrifugation at 7,822 × *g* for 15 min in a Sorvall Lynx 6000 centrifuge. The pellet was resuspended in 20 mM Tris buffer and frozen at −20°C. To the thawed cells, lysozyme (200 µg/mL final), DNase (50 µg/mL final), RNase (50 µg/mL final), and PMSF (1 mM final) were added. The cell suspension was sonicated for 9 min (amplitude = 40, 45 s on, 45 s off). Cell debris was pelleted by centrifugation at 41,743 × *g* (Sorvall Lynx 6000). The supernatant was first applied to a Q-Sepharose Fast Flow column (Cytiva), equilibrated in 20 mM Tris, pH 7.4. Scaffolding protein-containing fractions were pooled and dialyzed against 20 mM sodium phosphate buffer, pH 7.4. The dialyzed sample was then applied to an SP-Sepharose Fast Flow column (Cytiva), equilibrated in 20 mM sodium phosphate buffer, pH 7.4. Scaffolding protein-containing fractions were concentrated using 4 mL, 10 kDa MWCO centricons (MilliporeSigma) and applied to a Superdex 200 26/60 gel filtration column (Cytiva), equilibrated in 20 mM sodium phosphate buffer, pH 7.4. Scaffolding protein fractions were concentrated as above, and aliquots were stored until use at −80°C.

### Scaffolding protein-mediated *in vitro* ring oligomerization

WT and mutant portal protein monomers (16.5 µM) were incubated with scaffolding protein (135 µM) for 4 h at room temperature in 20 mM HEPES, 70 mM KAc buffer. The reaction was applied to a 5%–20% sucrose gradient, and the ring-containing fractions determined by SDS-PAGE were selected for analysis by negative stain TEM (8).

### Secondary structure determination by circular dichroism

Purified portal monomers or rings were diluted to 0.2 mg/mL in distilled water in a 0.1 cm quartz cuvette. CD spectra were collected using a Chirascan V100 spectrometer (Applied Photophysics, Leatherhead, UK) from 200 to 260 nm at 20°C with 1 nm intervals, a bandwidth of 1 nm, and time-per-point averaging of 5 s. Thermal denaturation of monomers and rings was performed by collecting spectra from 25°C to 80°C in 1°C steps, 1 nm intervals, 1 nm bandwidth, and time-per-point averaging of 1 s, and dwell time of 30 s.

### Proteolytic digest of rings by α-chymotrypsin

WT and variant portal rings were diluted to 0.11 mg/mL in portal dialysis buffer and digested with 0.0005 U/mL of α-chymotrypsin (Sigma, St. Louis, MO, USA) at 37°C. At time points from 0 to 120 min, aliquots were taken, added to SDS sample buffer containing 20 mM PMSF, and heated at 90°C for 5 min. The samples were run on a 10% SDS-PAGE gel to assess the cleavage patterns.

## Data Availability

All data needed to evaluate the results are included in the article.
